# Circulating cytokines in predicting development of severe acute pancreatitis

**DOI:** 10.1186/s13054-014-0575-0

**Published:** 2014-10-20

**Authors:** Ori D Rotstein

**Affiliations:** Keenan Research Centre for Biomedical Research, St Michael’s Hospital, 30 Bond Street 16cc-044, Toronto, ON M5B 1W8 Canada

## Abstract

Acute pancreatitis is an inflammatory disease process which may present with clinical manifestations ranging from benign self-limited disease to overwhelming sepsis. The ability to predict outcome would be helpful in developing treatment plans, and possibly in stratifying patients for clinical trials.

In a recent issue of *Critical Care*, Nieminen and colleagues [1] hypothesized that cytokine profiles might help predict disease severity and progression in patients presenting with acute pancreatitis. Acute pancreatitis is an inflammatory disease whose clinical presentation exhibits a broad spectrum of severity ranging from mild self-limited disease to severe progressive disease with organ dysfunction and often death. The Atlanta Classification defines three grades of severity based on clinical criteria, namely mild pancreatitis, moderately severe pancreatitis, and severe pancreatitis [2]. Mild pancreatitis shows no evidence of organ dysfunction and no local or systemic complications; moderately severe pancreatitis exhibits local or systemic complications with either no or transient organ dysfunction; severe pancreatitis is characterized by persistent single or multiorgan dysfunction. Whereas mild pancreatitis is relatively easy to sort out, the latter two entities are somewhat difficult. Various approaches to making this distinction have been investigated, including biochemical markers such as C-reactive protein, calcium, and creatinine; scoring systems such as Acute Physiology and Chronic Health Evaluation II; and radiological findings such as contrast-enhanced computerized tomography scans.

Evaluation of cytokine levels as predictors of disease severity is not entirely new, as several other investigators have studied the correlation between cytokine levels and the development of severe acute pancreatitis. IL-6, IL-8, and IL-10 were suggested as potential predictors [3]. In the present study, however, the authors have broadened the net of cytokines studied by using commercially available kits which can measure multiple cytokines (in this case, 48 different cytokines). There are several interesting and novel questions asked by the investigators in addition to just what cytokines are elevated. Specifically, they perform their measurements when the patient is admitted to the hospital with the diagnosis, asking whether the single data point correlates with severe pancreatitis. Second, they studied whether the cytokine profile can predict the progression of organ dysfunction in patients. This latter determination is performed in a retrospective fashion, since one would have only determined the development of organ dysfunction during the course of the hospital stay.

The study reports on a total of 163 patients, 25 of whom (15%) had a diagnosis of severe pancreatitis. Of this group, 11 had organ dysfunction on admission and 14 developed organ dysfunction within a week of admission. The major findings were that 14 out of 47 cytokines were higher on admission in patients who developed severe pancreatitis compared with those with moderately severe or mild disease and that IL-6 and hepatocyte growth factor (HGF) were independent predictors of severe acute pancreatitis. The positive likelihood ratio (+LR), a measure of diagnostic accuracy, was in the 5 to 10 range for each of these independently and in combination. A +LR in excess of 10 is interpreted as being consistent with a high likelihood of disease and therefore of use in clinical practice. It is noteworthy that some parameters currently used to predict severity - C-reactive protein, calcium, and creatinine - exhibited comparable +LR values. In the interesting group of patients who progressed to severe pancreatitis (that is, developed persistent organ dysfunction) during their hospital stay, IL-8, HGF, and granulocyte colony-stimulating factor were significantly higher than in those who did not. The low numbers of these patients precluded multivariate analysis. However, when the clinically optimal cutoff levels determined for each of these in the larger studies were used, the ability to predict was generally poor.

The authors are quite clear in stating that determination of the above-mentioned cytokines, either alone or in combination, at the time of admission does not add significantly to the prediction of clinical course in this patient population. This is perhaps not a surprising conclusion. Pancreatitis is a complex and heterogeneous disease which evolves over time. In the present article, subjects are included if they are admitted within 72 hours of the onset of their symptoms. We know from prior studies that IL-6 levels in pancreatitis can rise and fall over this time frame and thus the single admission time point may not be representative of the IL-6 response [4]. In the present article, the authors conclude that the discovery and measurement of other markers might improve the prediction of severe pancreatitis. Relevant to cytokine biomarkers, a recent translational research study implicated a role for soluble IL-6 receptor (sIL-6R) in mediating the pro-inflammatory effect of IL-6 during pancreatitis through a process called IL-6 trans-signaling, in which IL-6 complexes with sIL-6R and can induce alternative cellular signaling cascades [5]. The ratio of serum IL-6 to sIL-6R was suggested to be useful in distinguishing mild pancreatitis from severe pancreatitis with acute lung injury. Obviously, these and other novel pathways are worthy of study.

Finally, one might suggest that there is no need for studies such as those reported by Nieminen and colleagues, since there is no intervention which has been shown to modify the progression or severity of pancreatitis. However, practically speaking, when patients arrive with acute pancreatitis, it would be helpful to know whether this will progress or resolve. If the former, then closer observation in a monitored setting, consultation with a clinical expert, or even transfer to a specialty institution might be considered. Furthermore, having the ability to more accurately predict outcome would potentially permit stratification of patients for inclusion in clinical trials.

## Abbreviations

+LR: Positive likelihood ratio
HGF: Hepatocyte growth factor
IL: Interleukin
sIL-6R: Soluble interleukin-6 receptor

## References

[1] Nieminen A, Maksimow M, Mentula P, Kyhälä L, Kylänpää L, Puolakkainen P, Kemppainen E, Repo H, Salmi M (2014). Circulating cytokines in predicting development of severe acute pancreatitis. Crit Care.

[2] Banks PA, Bollen TL, Dervenis C, Gooszen HG, Johnson CD, Sarr MG, Tsiotos GG, Vege SS, Acute Pancreatitis Classification Working Group (2013). Classification of acute pancreatitis-2012: revision of the Atlanta classification and definitions by international consensus. Gut.

[3] Zhang J, Niu J, Yang J (2014). Interleukin-6, interleukin-8 and interleukin-10 in estimating the severity of acute pancreatitis: an updated meta-analysis. Hepatogastroenterology.

[4] Leser HG, Gross V, Scheibenbogen C, Heinisch A, Salm R, Lausen M, Rückauer K, Andreesen R, Farthmann EH, Schölmerich J (1991). Elevation of serum interleukin-6 concentration precedes acute-phase response and reflects severity in acute pancreatitis. Gastroenterology.

[5] Zhang H, Neuhöfer P, Song L, Rabe B, Lesina M, Kurkowski MU, Treiber M, Wartmann T, Regnér S, Thorlacius H, Saur D, Weirich G, Yoshimura A, Halangk W, Mizgerd JP, Schmid RM, Rose-John S, Algül H (2013). Il-6 trans-signaling promotes pancreatitis-associated lung injury and lethality. J Clin Invest.

